# First Genomic Evidence of California Hare Coltivirus from Natural Populations of *Ixodes persulcatus* Ticks in Northeast China

**DOI:** 10.3390/pathogens13080614

**Published:** 2024-07-25

**Authors:** Zhenyu Hu, Jingtao Zhang, Yantao Liu, Liming Liu, Fang Tang, Guangqian Si, Meiqi Zhang, Shuang Li, Yunfa Zhang, Cong Peng, Lei Zhang, Xiaofang Ma, Xiaoai Zhang, Wei Liu

**Affiliations:** 1 School of Public Health, Anhui Medical University, Hefei 230032, China; h19980706@163.com; 2 State Key Laboratory of Pathogen and Biosecurity, Academy of Military Medical Science, Beijing 100071, China; zjt66882022@yeah.net (J.Z.); qian857602721@163.com (G.S.); ic577703@163.com (M.Z.); zx2577102876@163.com (S.L.); fireflyzyf@126.com (Y.Z.); 13087737639@163.com (C.P.); zl632892@126.com (L.Z.); 3 Qingdao Municipal Center for Disease Control and Prevention, Qingdao 266033, China; lyt0102@sina.com (Y.L.); xf_mal@163.com (X.M.); 4 College of Animal Science and Technology, Jilin Agricultural Science and Technology University, Jilin 132101, China; aliuliming1984@126.com; 5 Institute of Medical Prevention and Control of Public Health Emergencies, Characteristic Medical Center of the Chinese People’s Armed Police Force, Beijing 102613, China; tf4065@163.com

**Keywords:** California hare coltivirus, *Ixodes persulcatus*, tick, coltivirus, China

## Abstract

**Background:** California hare coltivirus (CHCV) was isolated in California in 1976 from a hare. Despite its long history, it remained unclear whether CHCV was exclusively distributed in California with limited host ranges. **Main body:** By next-generation sequencing (NGS), we obtained a complete sequence of CHCV from *Ixodes persulcatus* collected in 2019 in northeast China. An expanded epidemiological investigation was subsequently performed on ticks belonging to four species (*Ix. persulcatus*, *Haemaphysalis concinna*, *Devmacentor silvarum*, *Haemaphysalis longicornis*) collected in northeastern China by applying CHCV-specific RT-PCR and sequencing. CHCV RNA-positive results were found in 1.56% of the tick samples. Positive ticks were obtained in three of four sampled locations, with the highest rate observed in Inner Mongolia (2.69%), followed by Heilongjiang (1.94%) and Jilin provinces (0.55%). All positive results were derived from *Ix. persulcatus* ticks (2.33%), while no positive detection was found in the other tick species, even at the same location. Sequence analysis revealed that the current CHCV showed a high genetic identity (>80% amino acid identity) with the previously reported CHCV in all segments except segment seven (64.59% amino acid identity). Phylogenetic analysis based on RNA-dependent RNA polymerase (RdRp) amino acid sequences demonstrated that both the current and previously reported CHCV strains were grouped phylogenetically into the genus *Coltivirus*. Both CHCV strains formed a distinct clade, clustering with three human pathogenic coltiviruses (Colorado tick fever virus, Salmon River virus, and Eyach virus), and were distant from the other coltiviruses. **Conclusions:** We report the identification and characterization of CHCV for the first time in *Ix. persulcatus* ticks, expanding the currently known geographic scope, host, and genetic heterogeneity in CHCV.

## 1. Introduction

**Figure 1:**
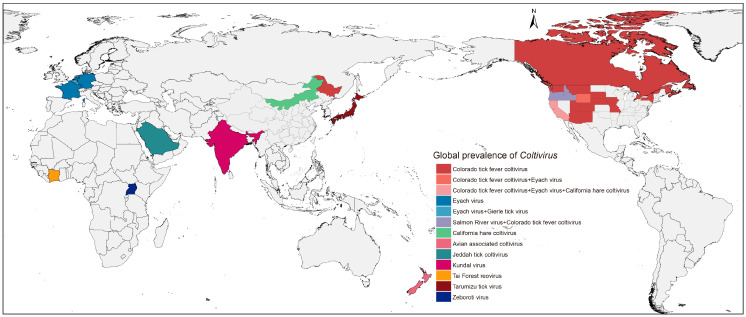
The global distribution of coltiviruses.

The family *Spinareoviridae* is a group of icosahedral viruses classified into nine genera (https://ictv.global/taxonomy/; accessed on 20 December 2023). *Spinareoviridae* viruses are typically considered non-enveloped, with segmented double-stranded (ds) RNA genomes (9–12 linear segments) totaling 23–29 kbp. Viruses within this family possess spikes protruding from the outer capsid layer, distinguishing them from their sister family *Sedoreoviridae*. Within the *Spinareoviridae* family, the genus *Coltivirus* has an extensive history and global distribution among various animal species (Figure 1). Coltiviruses also include major species responsible for human infections including Colorado tick fever virus (CTFV) and Salmon River virus (SARV) [1,2]. CTFV was first isolated in 1943 from a febrile patient and has since been found to be prevalent in the Rocky Mountain region of North America, where its vector, *Dermacentor andersoni*, is common [3]. Another member, SARV, was first isolated from an individual with illness in Idaho in 1990 [1]. In addition to CTFV, other members within the genus *Coltivirus* such as Eyach virus (EYAV), Kundal virus, Tarumizu tick virus (TarTV), Gierle tick virus, and Jeddah tick coltivirus, have been isolated or detected from ticks across Europe, India, Japan, Belgium, and Saudi Arabia, respectively [4,5,6,7]. Tai Forest reovirus (TFRV) was isolated from free-tailed bats captured in Côte d’Ivoire in 2006, while California hare coltivirus (CHCV) was detected in rabbits in the USA [8]. However, despite recent progress in the understanding of coltiviruses, where and how they are maintained in nature remains unclear and is an actively pursued and debated area of research. Among them, as a scarcely investigated virus, CHCV was only reported in lepus californicus (black-tailed jackrabbit) specimens in 1976. There is a lack of information regarding vector, distribution, molecular evolution, and data on the control method for this virus, which hinders an accurate description of the viral evolution and epidemiology. The aim of this study was to present the characterization and epidemiology of CHCV in ticks in northeast China.

## 2. Materials and Methods

### 2.1. Study Site and Sample Collection

**Figure 2:**
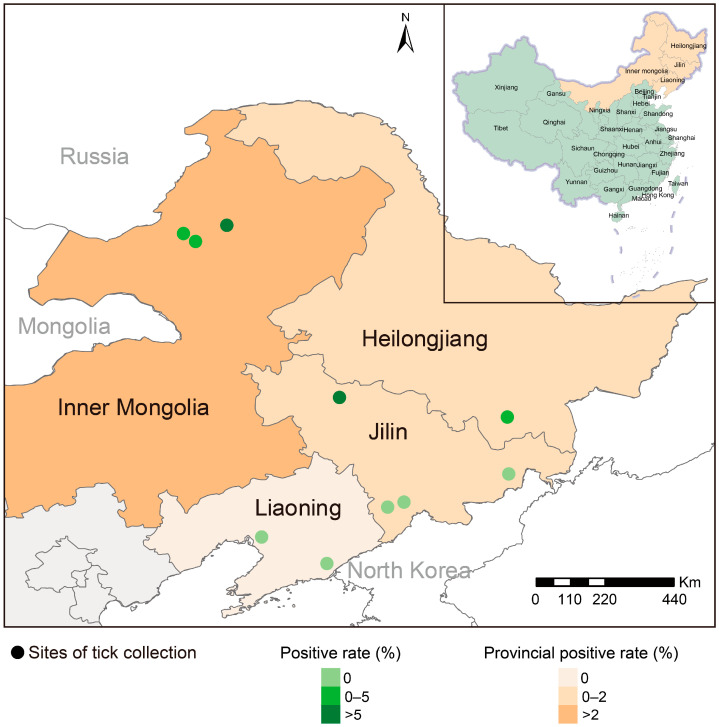
Geographic distribution of California hare coltivirus (CHCV) in northeastern China. Areas where ticks were collected are shown in orange. The green color of the circles indicates the positive rate of CHCV in ticks; the orange color represents the positive rate of CHCV in the area.

From April 2019 to June 2023, free-living ticks were collected by flagging over vegetation from four provinces (Autonomous Region), i.e., Inner Mongolia (120°42′53″–121°36′29″ E, 49°17′29″–50°1′35″ N), Heilongjiang (129°37′33″ E, 44°32′59″ N), Jilin (124°49′2″–130°21′33″ E, 41°43′34″–45°8′19″ N), and Liaoning (122°14′9”–124°22′56″ E, 40°7′26″–40°22′60″ N), during an ongoing program aimed at identifying pathogens in ticks in China (Figure 2). Ticks were collected with the standard tick-dragging method. The tick species were initially identified based on morphology [9,10], and further confirmed through PCR–Sanger sequencing of mitochondrial 16S ribosomal DNA (16S rDNA) [11] (Table S1). All collected samples were stored at −80 °C prior to use.

### 2.2. Extraction of Viral RNA and Next-Generation Sequencing (NGS)

Sixty randomly selected adult ticks from two dominant tick species (*Ixodes persulcatus* and *Haemaphysalis concinna*) in the Inner Mongolia Autonomous Region were subjected to next-generation sequencing (NGS) as previously described [12]. Briefly, total RNA was extracted using the AllPrep DNA/RNA Mini Kit (Qiagen, Hilden, Germany), from which rRNA was removed using the MGIEasy rRNA Depletion Kit (BGI, Shenzhen, China). A high-throughput sequencing library was constructed using an MGIEasy RNA Library Prep Kit (BGI). Viral gene libraries were then sequenced using the MGI2000 platform (BGI). After processing the raw data through filtering, trimming, and error removal steps, the remaining reads were aligned to NCBI viral reference genome sequences utilizing Bowtie2 (v2.3.5.1). De novo assembly was performed with MEGAHIT (v1.2.9), and the assembled contigs were compared against the NCBI non-redundant protein database using Diamond BLASTX (v2.0.14.152). Virus-associated contigs were mapped to their corresponding reference sequences.

### 2.3. Real-Time RT-PCR for CHCV

CHCV screening was performed by PCR amplification of a 136 bp fragment of the VP12 gene by using specific primers and probes designed according to the current CHCV (GenBank accession PP444695) and the previously reported CHCV (GenBank accession MW396991.1). A One-step Primer Script RT-PCR Kit (TaKaRa Biotech, Dalian, China) was used according to the manufacturer’s instructions for CHCV detection. The sequences of qRT-PCR primers and probe were as follows: forward: 5′ATGTMTCRAAGACMTCACTTC-3′; reverse: 5′GTCTCATATACGTGAGCAAYG-3′; Probe: 5′FAM-GWSTTGACCGAGATCCAGGA-MGB-3′. The PCR mix was in a volume of 20 µL containing 10 µL of One-Step RT-PCR Buffer (2×), 0.4 µL of TaKaRa Ex Taq HS (5 U/µL), and 0.4 µL of PrimeScript^TM^ RT Enzyme MixII, 1 µL of PCR primer mix (20 µM of sense and antisense each) and 0.5 µL of Probe (10 µM), total RNA 2 µL, and RNase free dH2O (5.7 µL). PCR was carried with one cycle of 42 °C for 5 min and 95 °C for 30 s, followed by 40 cycles of 95 °C for 5 sec and 55 °C for 35 s in the Light Cycler 480 Real-Time PCR System (Roche, Indianapolis, IN, USA). All steps of the nucleic acid extraction and PCR test were conducted in parallel with positive (RNA from the positive sample) and negative controls (RNase-free water). The prevalence (95% confidence intervals) was calculated by maximum likelihood estimation (MLE) using the program PooledInfRate (www.cdc.gov/mosquitoes/php/toolkit/mosquito-surveillance-software.html; accessed on 29 May 2024).

### 2.4. Phylogenetic Analysis

Amino acid sequences from the currently detected CHCV and other representative species from the family *Spinareoviridae* were downloaded from GenBank and aligned by the ClustalW method using MEGA-X (Tables S2 and S3). Phylogenetic trees were established using the maximum likelihood method by the best-fitting model, which was determined by the ModelFinder program implemented in IQ-TREE (v1.6.12). Bootstrap values were calculated based on 1000 replicates. Potential recombination events within viral genomes and possible recombination breakpoints were identified through Simplot (v3.5.1) [13] and RDP (v4.97) analysis [14]. The sequences generated in this study were submitted to GenBank under accession numbers PP444695–PP444706.

### 2.5. Relationship between Coltiviruses and Host

Host range determination relied on host species information retrieved from NCBI and supplemented with the newly identified hosts in this study. Briefly, all nucleotide sequences of 11 coltiviruses of the genus *Coltivirus* were retrieved from the NCBI/GenBank (https://ftp.ncbi.nlm.nih.gov/genbank; accessed on 15 December 2023), and detailed information (including references, sampling locations, host, and collection date) was collected. Further details were obtained from the references if the original virus sequence information was incomplete. We collected and organized the necessary sequence information and then, using the R “networkD3” package (v0.4), plotted Sankey plots for the relationship between coltiviruses and hosts.

## 3. Results

### 3.1. Identification of CHCV in Ha. concinna by NGS

**Figure 3:**
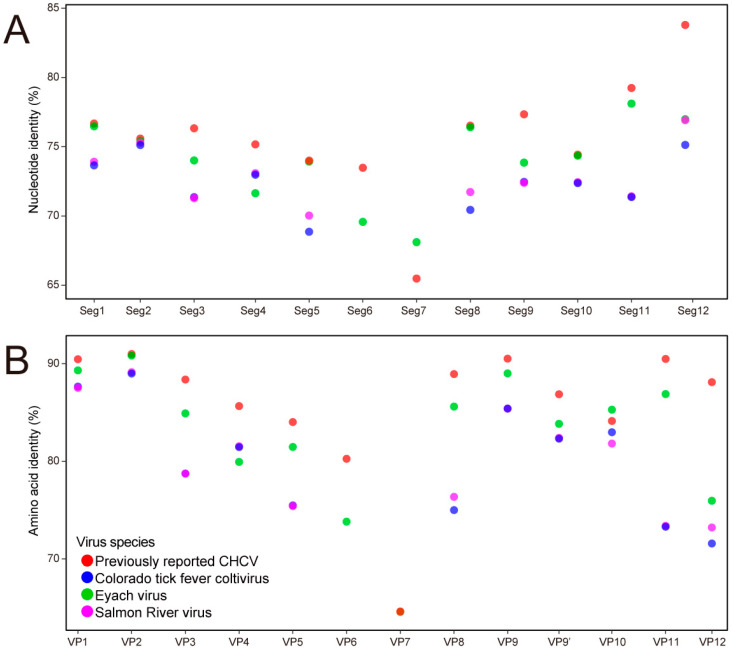
Nucleotide similarity (**A**) and amino acid similarity (**B**) among the current CHCV, the previously reported CHCV, and three closely related species in the genus *Coltivirus*, CTFV, EYAV, and SARV.

After metagenomic analysis of two dominant tick species, specific sequences of CHCV were identified from *Ix. persulcatus* collected in 2019 in the Inner Mongolia Autonomous Region. Out of a total of 191,886,372 reads obtained, 1936 were annotated to CHCV. No CHCV-specific sequence was found from *Ha. concinna* using NGS. The matched sequences of CHCV could be assembled into 12 nearly complete segments (Figure S1). Percent identities comparing nucleotides (Figure 3A) and amino acids (Figure 3B) were calculated for the current CHCV, the previously reported CHCV, and the three closely related species in the genus *Coltivirus*, CTFV, EYAV, and SARV (Figures S2–S14). The full-length amino acid sequences of the current CHCV showed high genetic identity (>80% amino acid identity) with the previously reported CHCV in all segments except segment seven (64.59% amino acid identity). The current CTFV exhibited high nucleotide variability compared with CTFV, EYAV, and SARV across all segments; however, segment seven displayed the highest genetic diversity (Figure 3A and Table S4). Overall, the percent identities between the current CTFV and the three closely related species, CTFV, EYAV, and SARV, at the amino acid level ranged from 90.63% amino acid identity in VP2 to 64.59% in VP7, all higher compared with the identities at the nucleotide level except VP7 and VP12 (Figure 3B and Table S5).

### 3.2. Phylogenetic Analysis of CHCV Sequences

**Figure 4:**
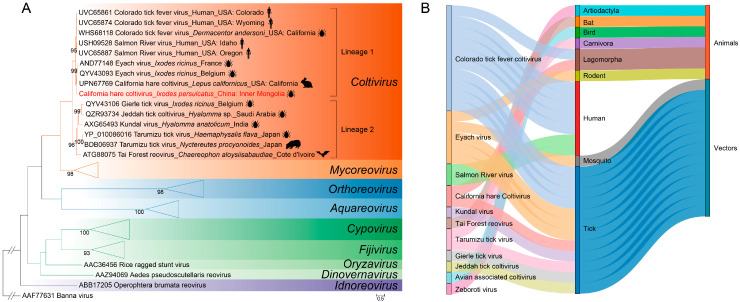
(**A**) The maximum likelihood phylogenetic tree constructed based on the amino acid sequences of RdRp (VP1) in Spinareoviridae. Phylogenetic inference was performed using the maximum likelihood (ML) method with 1000 bootstrap replicates. Branch lengths are indicated by the scale bar. (**B**) A tripartite network visualizing the associations among recognized viruses in the genus *Coltivirus* and their potential vectors and hosts using a combination of our data and the virus records from the NCBI/GenBank database.

The RNA-dependent RNA polymerase (RdRp) amino acid sequences from our CHCV virus and nine recognized species in the genus *Coltivirus* were subjected to phylogenetic analysis. The results revealed that both the current and previously reported CHCV strains were grouped phylogenetically into the genus *Coltivirus.* They formed a distinct clade, clustering with three human pathogenic coltiviruses (CTFV, SARV, and EYAV), and were distant from the other coltiviruses (Figure 4A). Similar phylogenetic trees were inferred for all segments, except VP6 and VP10 (Figure S15). In the VP6 phylogenetic tree, the current CHCV and previously reported CHCV clustered with EYAV, while in the VP10 phylogenetic tree, all strains of CHCV clustered not only with human pathogenic coltiviruses, CTFV, SARV, and EYAV, but also with TFRV isolated from free-tailed bats captured in 2006 (Figure S15). No recombination events involving the current CHCV, the previously reported CHCV, or CTFV, SARV, and EYAV were detected across the 12 genome sequences analyzed (Figure S16). We also constructed a Sankey plot linking host models of the genus *Coltivirus* by collecting host information for all recognized species from the NCBI/GenBank database. These coltiviruses are distributed among nine different orders of hosts, ticks being the most common host groups, accounting for seven out of eleven viruses. Furthermore, RNA of CTFV and SARV has been detected in humans as well (Figure 4B).

### 3.3. CHCV Screening in Ticks by Real-Time RT-PCR

A molecular epidemiologic study was conducted to assess the diversity of CHCV in the ticks captured in northeast China. A total of 872 ticks belonging to four species (*Ix. persulcatus*, *Ha. concinna*, *De. silvarum*, *Ha. longicornis*) were collected, which were further grouped into 363 pools by species and locations for further testing. The overall prevalence of CHCV detection in ticks was determined to be 1.56% (0.87–2.59%) (Table 1). Positive ticks were obtained in three of four sampled regions, with the highest rate observed in Inner Mongolia (2.69%), followed by Heilongjiang (1.94%) and Jilin provinces (0.55%) (Table 1). All positive results were derived from *Ix. persulcatus* ticks (2.33%), while no positive detection was found in other tick species, even at the same location.

## 4. Discussion

After its first emergence in California in 1976, it remained obscure whether CHCV was exclusively distributed in California with limited host ranges. In this study, by screening four tick species from northeast China, we determined the presence of CHCV in *Ix. persulcatus* ticks for the first time, indicating CHCV can circulate in northeast China.

The family *Spinareoviridae* currently encompasses RNA viruses from nine genera and 58 species (https://talk.ictvonline.org; accessed on 3 March 2024), among which five have been identified as causing human infection, three of which belong to the genus *Coltivirus* and are transmitted by ticks, distinguishing themselves from most other members within the *Spinareoviridae* family. Within the genus *Coltivirus*, apart from two major species responsible for human infections—CTFV and SARV—antibodies against EYAV have been identified in sera samples from patients with neurological syndromes, suggesting a potential involvement of EYAV in causing neuroinvasive disease in humans [15]. In this study’s phylogenetic inference analysis, CHCVs were consistently clustered together with three known human pathogenic coltiviruses across 11 of the 13 proteins examined. These tree topologies are supported by evolutionary distances indicating that CHCV is more closely related to human pathogenic coltiviruses than other coltiviruses. Although we did not succeed in isolating CHCV itself, its close phylogenetic relationship with other viruses known to infect humans does warrant further research into this virus as a potential human or animal pathogen.

CHCV was initially discovered in *lepus californicus* specimens collected from California in 1976. However, apart from epidemiology evidence from the USA, there is a lack of information regarding its distribution in other countries and data on the molecular evolution of this virus, which hinders an accurate description of the viral evolution and epidemiology. In this study, we report the identification and characterization of CHCV for the first time in adult ticks *Ix. persulcatus* found in China, further confirming its natural circulation among ticks in northeastern China.

*Ix. persulcatus* is known to inhabit various regions across the Eurasian continent, including Russia, China, Japan, and Estonia, within a latitude range of approximately 21–66° N [16]. In China specifically, *Ix. persulcatus* is most prevalent in the northeast and northwest areas, such as Heilongjiang, Jilin, Liaoning, Inner Mongolia, and Xinjiang [16]. This tick species has been found to feed on 46 different host species and can harbor up to 51 tick-borne agents, posing a significant public health threat across northern Europe, western Russia, and northern China [16]. While CHCV prevalence has been confirmed only in Inner Mongolia and Jilin so far, the endemicity of CHCV should be investigated further in other regions where *Ix. persulcatus* is present. Serological investigations targeting domestic animals, wild animals, and humans should be conducted not only within Inner Mongolia and Jilin but also in other potential endemic regions to gain a comprehensive understanding of the transmission cycle and public health significance of CHCV infection.

## 5. Conclusions

CHCV, a tick-borne coltivirus, was identified in China, representing its first detection outside of the USA. The data presented herein highlight an underappreciated diversity of CTFV. Despite a relatively low prevalence of infection in *Ix. persulcatus*, our results call for further investigations including active surveillance and a phylogenetic approach to enhance our understanding of the epidemiological characteristics of CHCV and guide future human infection diagnosis and management. Further studies that combine active surveillance, viral isolation, and a phylogenetic approach might help to enhance our understanding of the epidemiological characteristics of this scarcely investigated virus and guide the diagnosis and management of its potential infection in human beings.

## Figures and Tables

**Table 1:** Detection of California hare coltivirus in ticks by real-time RT-PCR *.

Tick Species	Inner Mongolia	Heilongjiang	Jilin	Liaoning	Total
NPP/NTP (NTT)					
*Ixodes persulcatus*	10/59 (311)	2/100 (104)	1/41 (180)	nd	13/200 (595)
*Devmacentor silvarum*	0/48 (48)	nd	nd	nd	0/48 (48)
*Haemaphysalis concinna*	0/48 (48)	nd	nd	nd	0/48 (48)
*Haemaphysalis longicornis*	nd	nd	nd	0/67 (181)	0/67 (181)
Total	10/155 (407)	2/100 (104)	1/41 (180)	0/67 (181)	13/363 (872)
Prevalence (%, 95% CI)					
*Ixodes persulcatus*	3.62 (1.85–6.46)	1.94 (0.35–6.23)	0.55 (0.03–2.65)	nd	2.33 (1.30–3.88)
*Devmacentor silvarum*	0	nd	nd	nd	0
*Haemaphysalis concinna*	0	nd	nd	nd	0
*Haemaphysalis longicornis*	nd	nd	nd	0	0
Total	2.69 (1.36–4.81)	1.94 (0.35–6.23)	0.55 (0.03–2.65)	0	1.56 (0.87–2.59)

* The prevalence (95% confidence intervals) was calculated by maximum likelihood estimation using the program PooledInfRate. NPP, no. of positive pools; NTP, no. of tick pools; NTT, no. of tested ticks; nd, no data.

## Data Availability

The aggregate data supporting findings contained within this manuscript will be shared upon request submitted to the corresponding author.

## Supplementary Materials

The following supporting information can be downloaded at https://www.mdpi.com/article/10.3390/pathogens13080614/s1, Figure S1: Full-genome structure of four representative viruses (CHCV, CTFV, EYAV, and SARV); Figure S2: Amino acid alignment of VP1 between CHCV_China and the representative *Coltivirus* in *Spinareoviridae*; Figure S3: Amino acid alignment of VP2 between CHCV_China and the representative *Coltivirus* in *Spinareoviridae*; Figure S4: Amino acid alignment of VP3 between CHCV_China and the representative *Coltivirus* in *Spinareoviridae*; Figure S5: Amino acid alignment of VP4 between CHCV_China and the representative *Coltivirus* in *Spinareoviridae*; Figure S6: Amino acid alignment of VP5 between CHCV_China and the representative *Coltivirus* in *Spinareoviridae*; Figure S7: Amino acid alignment of VP6 between CHCV_China and the representative *Coltivirus* in *Spinareoviridae*; Figure S8: Amino acid alignment of VP7 between CHCV_China and the representative *Coltivirus* in *Spinareoviridae*; Figure S9: Amino acid alignment of VP8 between CHCV_China and the representative *Coltivirus* in *Spinareoviridae*; Figure S10: Amino acid alignment of VP9 between CHCV_China and the representative *Coltivirus* in *Spinareoviridae*; Figure S11: Amino acid alignment of VP9′ between CHCV_China and the representative *Coltivirus* in *Spinareoviridae*; Figure S12: Amino acid alignment of VP10 between CHCV_China and the representative *Coltivirus* in *Spinareoviridae*; Figure S13: Amino acid alignment of VP11 between CHCV_China and the representative *Coltivirus* in *Spinareoviridae*; Figure S14: Amino acid alignment of VP12 between CHCV_China and the representative *Coltivirus* in *Spinareoviridae;* Figure S15: Phylogenetic analysis of California hare Coltivirus_China for the deduced amino acid sequences (VP2-VP12); Figure S16: Similarity plot of California hare Coltivirus_China and other representative viruses in Seg1–Seg12. Table S1: Primers for identifying species of ticks; Table S2: Viruses in *Spinareoviridae* used for phylogenetic analysis based on the RNA-dependent RNA polymerase (RdRp) gene in this study; Table S3: Viruses in *Coltivirus* used for phylogenetic analysis based on the VP2–VP12 gene in this study; Table S4: Nucleotide similarity (%) between California hare Coltivirus_China and the representative *Coltivirus* in *Spinareoviridae*; Table S5: Amino acid similarity (%) between California hare Coltivirus_China and the representative *Coltivirus* in *Spinareoviridae*.
