# Supplementary material for: First Genomic Evidence of California Hare Coltivirus from Natural Populations of Ixodes persulcatus Ticks in Northeast China
Source: Pathogens. 2024 Jul 25;13(8):614. doi: 10.3390/pathogens13080614 (PMC11357685; doi:10.3390/pathogens13080614)
Supplement: Supplementary file 1 [file pathogens-13-00614-s001.zip › Supplemental Materials.pdf]

Materials and Methods

Real-time RT-PCR for CHCV

One step Primer Script RT-PCR Kit (TaKaRa) was used according to the manufacturer's instructions for CHCV detection. The sequences of qRT-PCR primers and probe were as follows: forward: 5′ATGTMTCRAAGACMTCACTTC -3′; reverse: 5′GTCTCATATACGTGAGCAAYG-3′; Probe: 5′FAM-GWSTTGACCGAGATCCAGGA-MGB-3′. The PCR mix was in a volume of 20 µl containing 10 µl of One Step RT-PCR Buffer (2×), 0.4 µl of TaKaRa Ex Taq HS (5 U/µl) and 0.4 µl of PrimeScript^{TM} RT Enzyme MixII, 1 µl of PCR primer mix (20 µM of sense and antisense each) and 0.5µl of Probe (10 µM), total RNA 2 µl and RNase free dH2O (5.7 µl). PCR was carried with one cycle of 42ºC for 5 min and 95ºC for 30 sec, followed by 40 cycles of 95ºC for 5 sec and 55ºC for 35 sec in Light Cycler 480 Real-Time PCR System (Roche, USA). All steps of the nucleic acid extraction and PCR test were conducted in parallel with positive and negative controls.

Figure S1. Full-genome structure of four representative viruses (CHCV, CTFV, EYAV, and SARV).

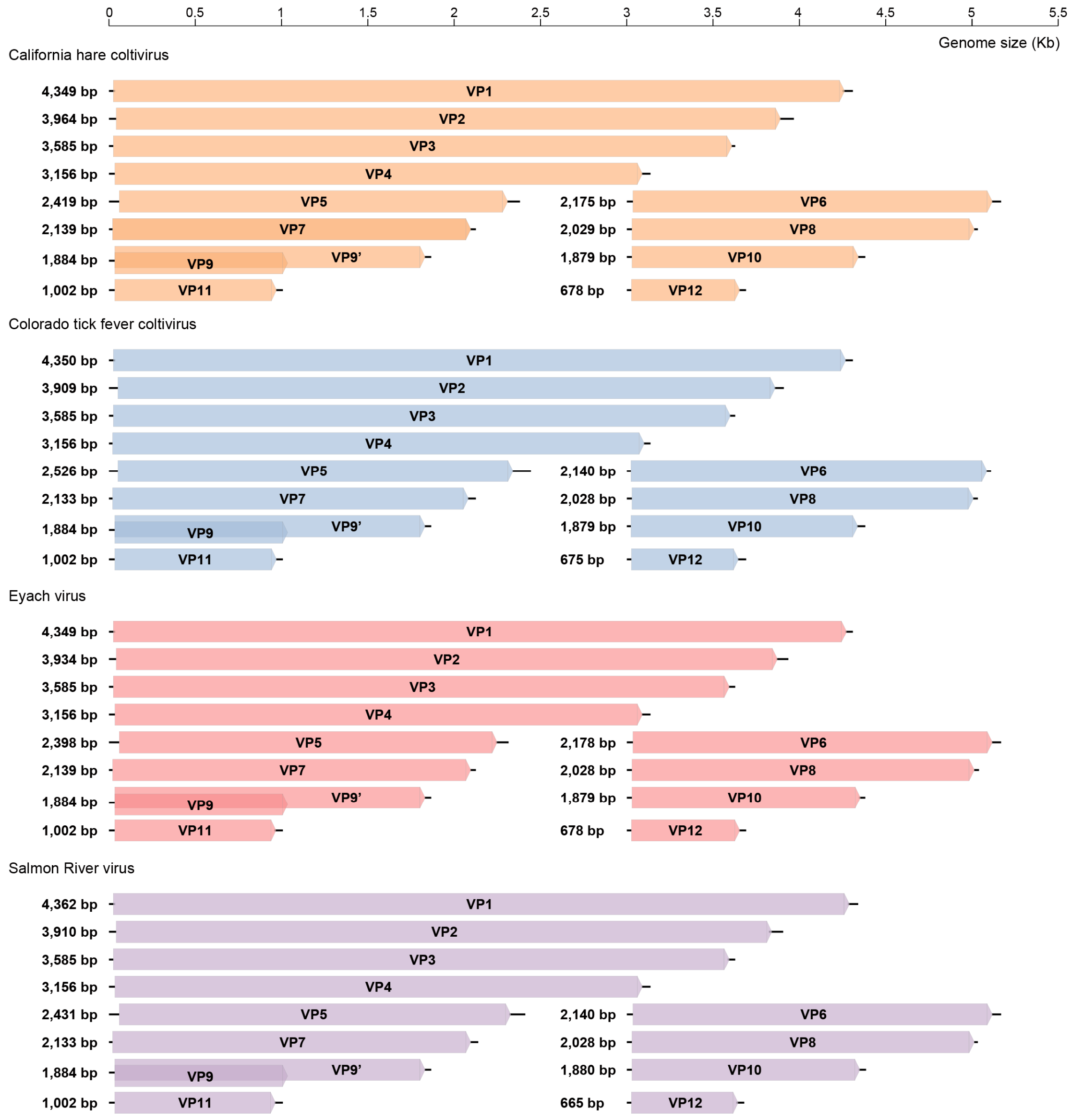

* CHCV, California hare coltivirus; CTFV, Colorado tick fever coltivirus; EYAV, Eyach virus; SARV, Salmon River virus.

Figure S2. Amino acid alignment of VP1 between CHCV_China and the representative *Coltivirus* in *Spinareoviridae*.

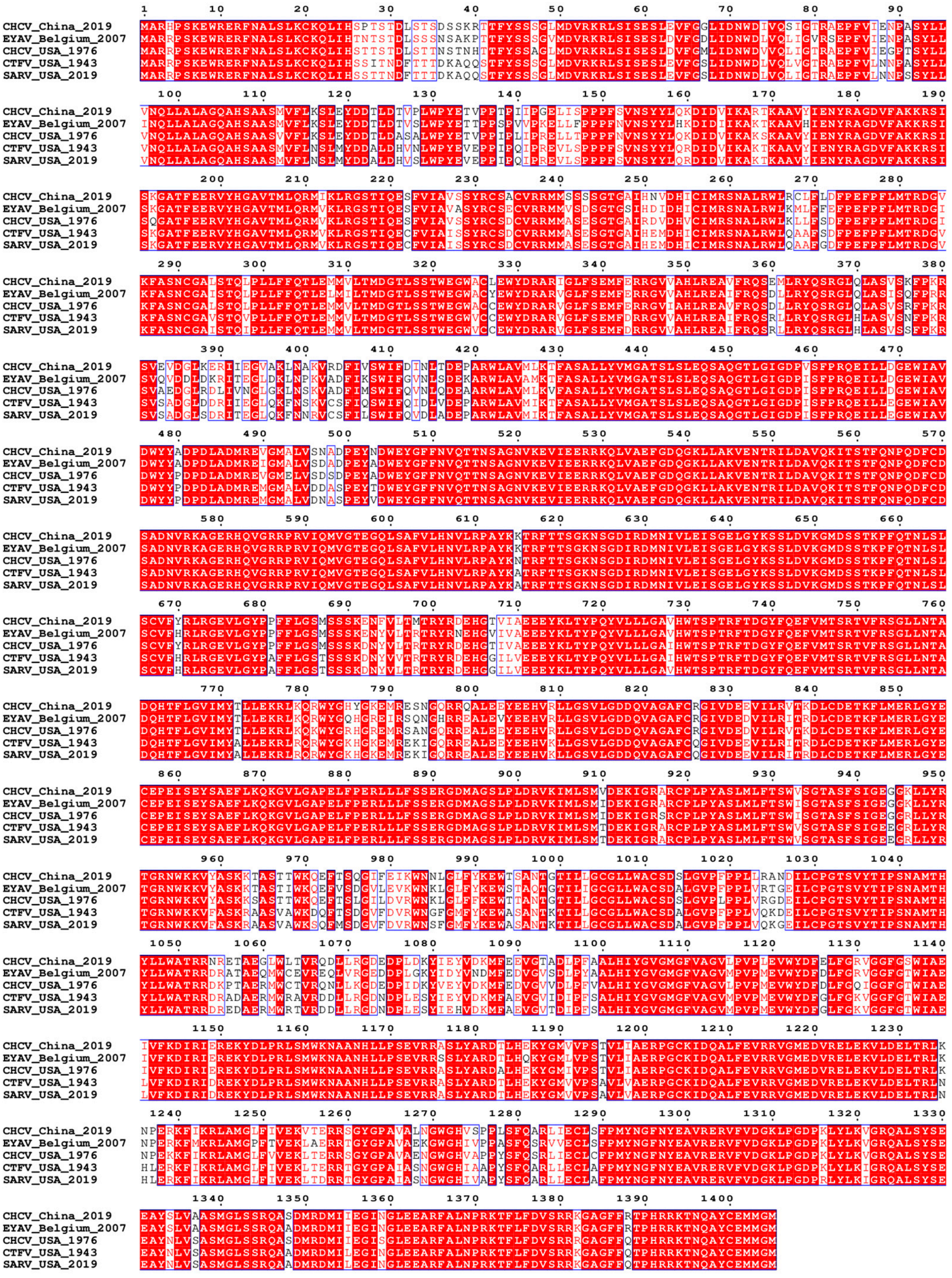

* CHCV, California hare coltivirus; CTFV, Colorado tick fever coltivirus; EYAV, Eyach virus; SARV, Salmon River virus.Amino acid alignment was performed using ESPript 3.0 (http://espript.ibcp.fr/ESPript/cgi-bin/ESPript.cgi)

Figure S3. Amino acid alignment of VP2 between CHCV_China and the representative *Coltivirus* in *Spinareoviridae*.

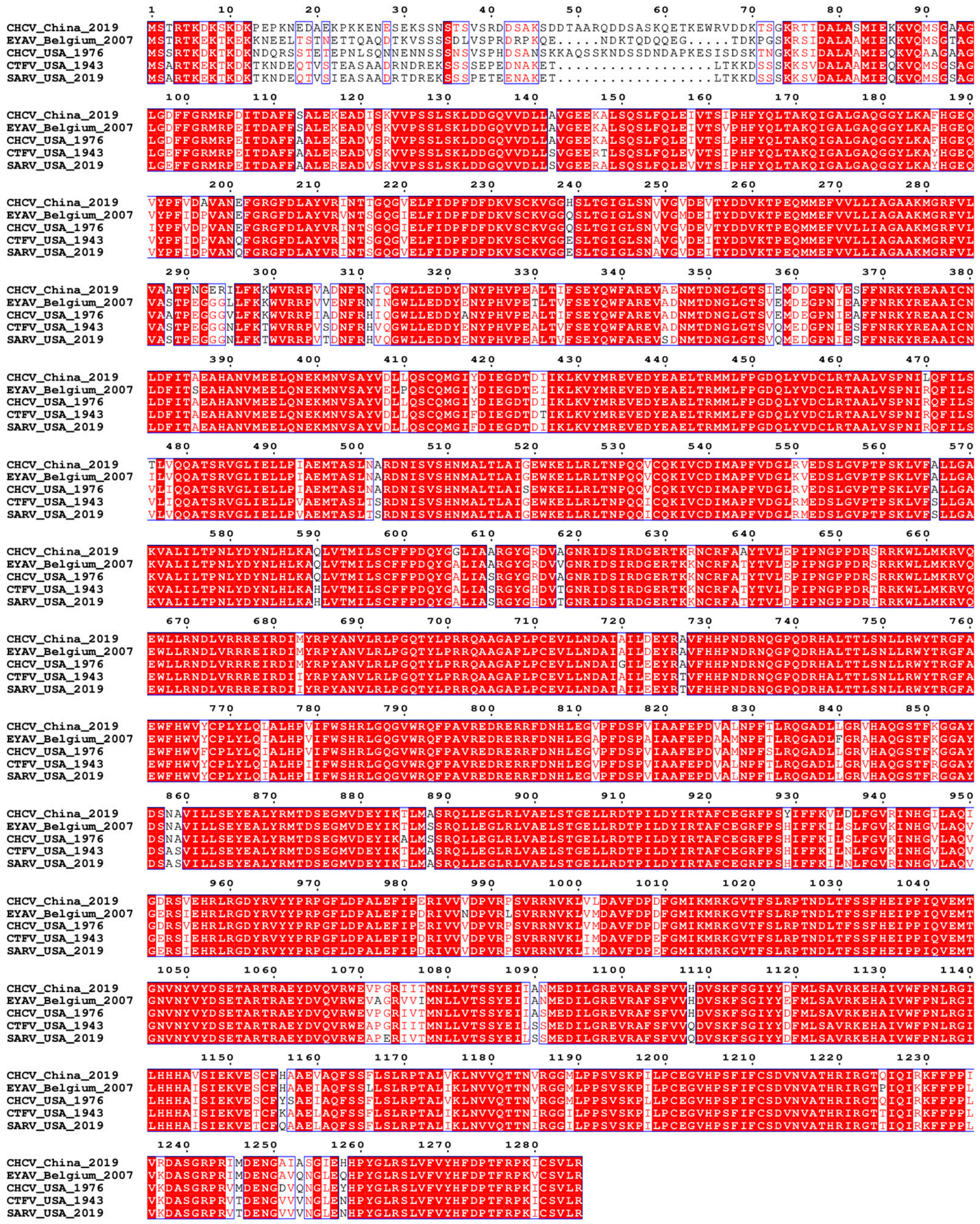

* CHCV, California hare coltivirus; CTFV, Colorado tick fever coltivirus; EYAV, Eyach virus; SARV, Salmon River virus.

Figure S4. Amino acid alignment of VP3 between CHCV_China and the representative *Coltivirus* in *Spinareoviridae*.

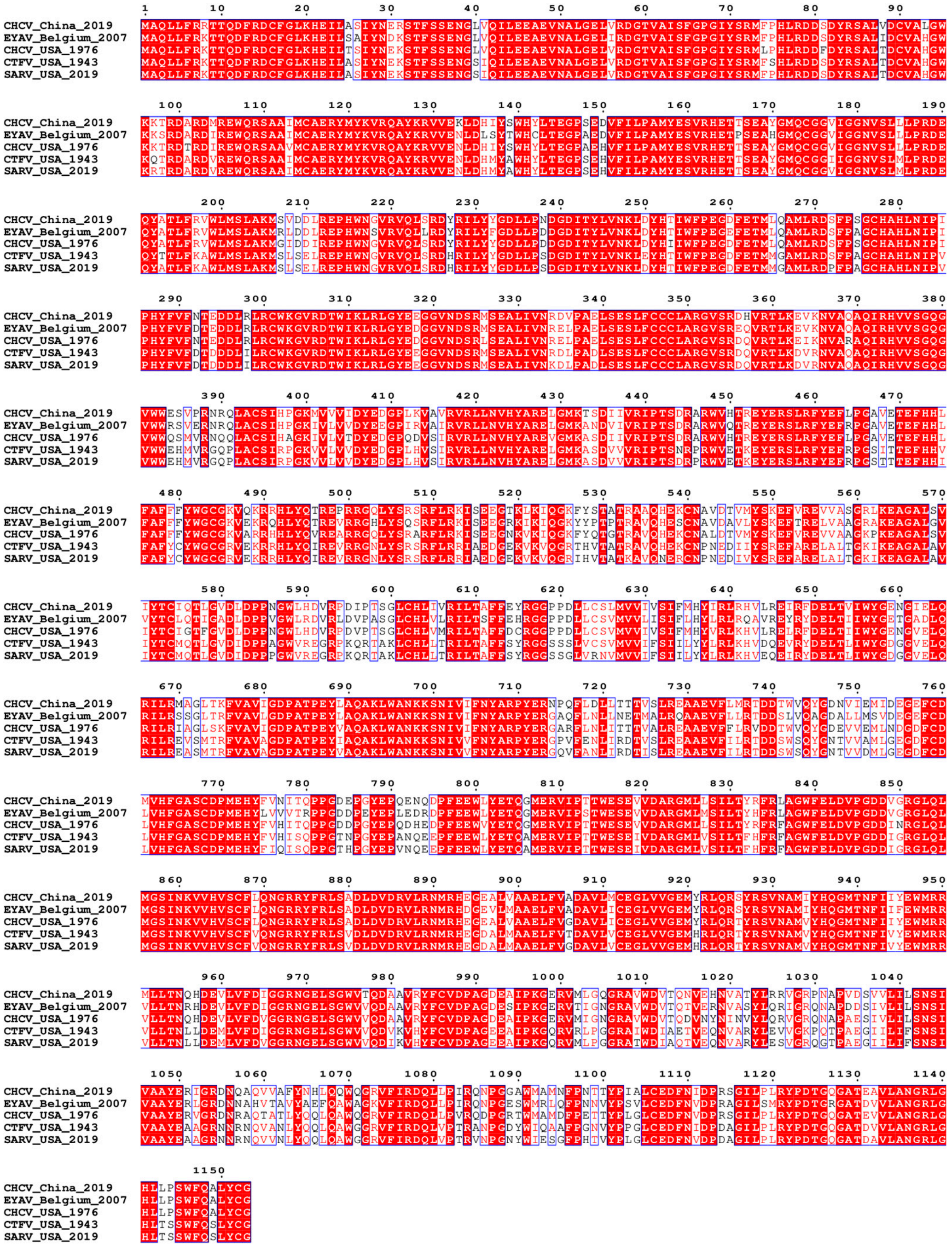

* CHCV, California hare coltivirus; CTFV, Colorado tick fever coltivirus; EYAV, Eyach virus; SARV, Salmon River virus.

Figure S5. Amino acid alignment of VP4 between CHCV_China and the representative *Coltivirus* in *Spinareoviridae*.

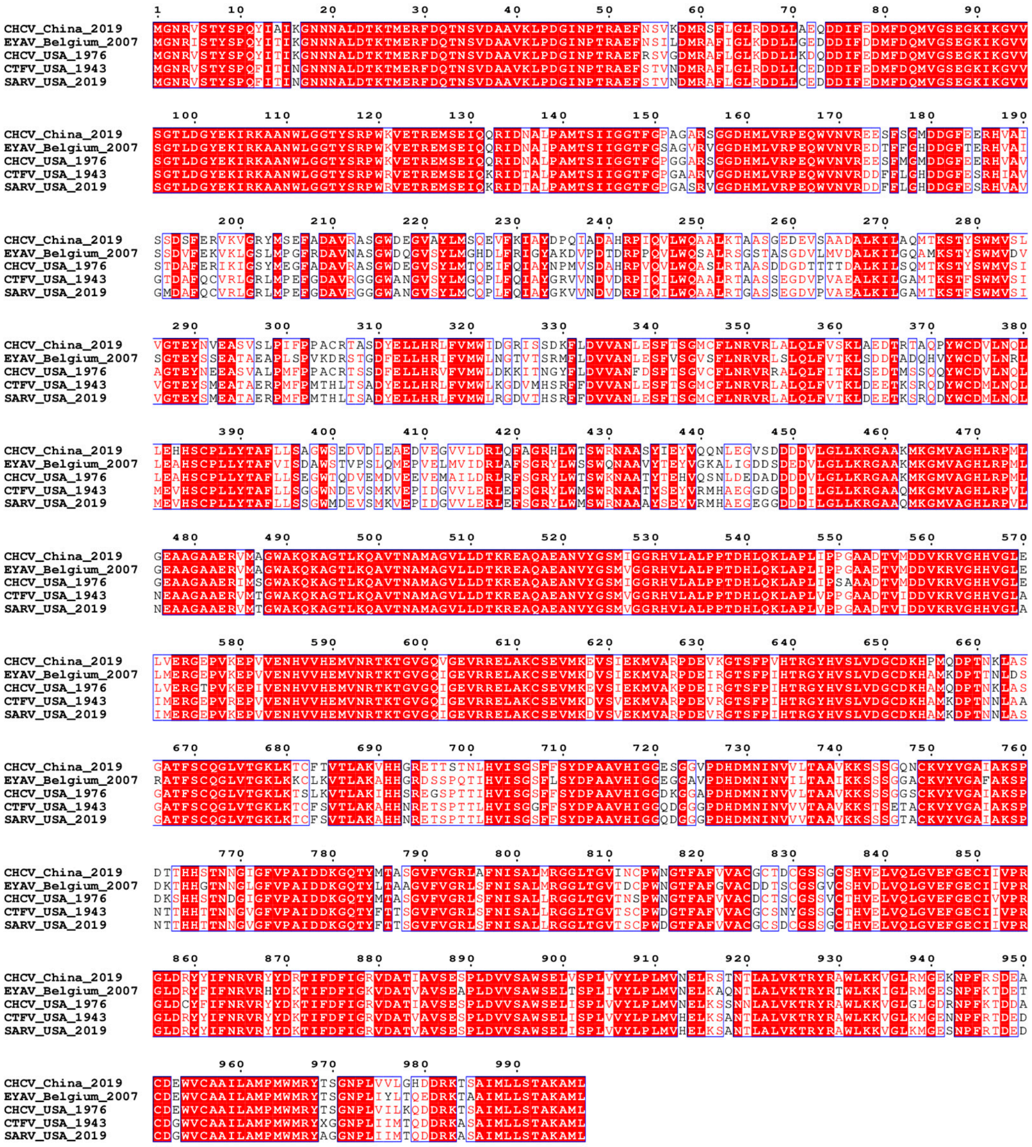

* CHCV, California hare coltivirus; CTFV, Colorado tick fever coltivirus; EYAV, Eyach virus; SARV, Salmon River virus.

Figure S6. Amino acid alignment of VP5 between CHCV_China and the representative *Coltivirus* in *Spinareoviridae*.

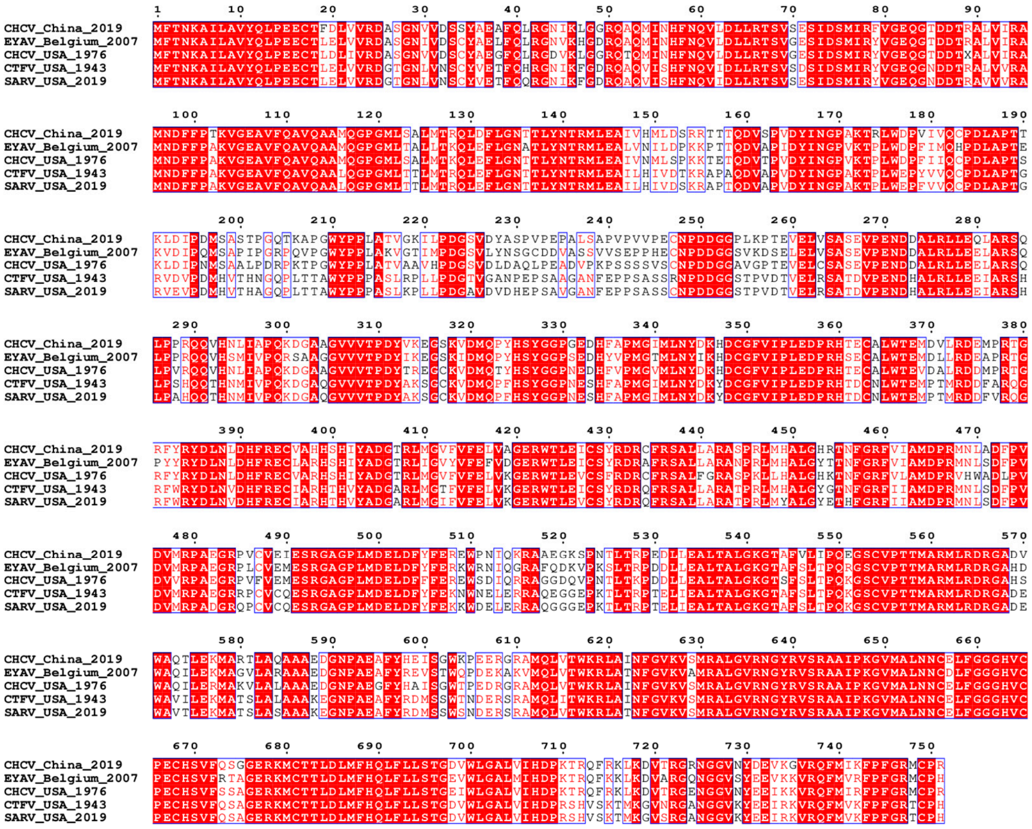

* CHCV, California hare coltivirus; CTFV, Colorado tick fever coltivirus; EYAV, Eyach virus; SARV, Salmon River virus.

Figure S7. Amino acid alignment of VP6 between CHCV_China and the representative *Coltivirus* in *Spinareoviridae*.

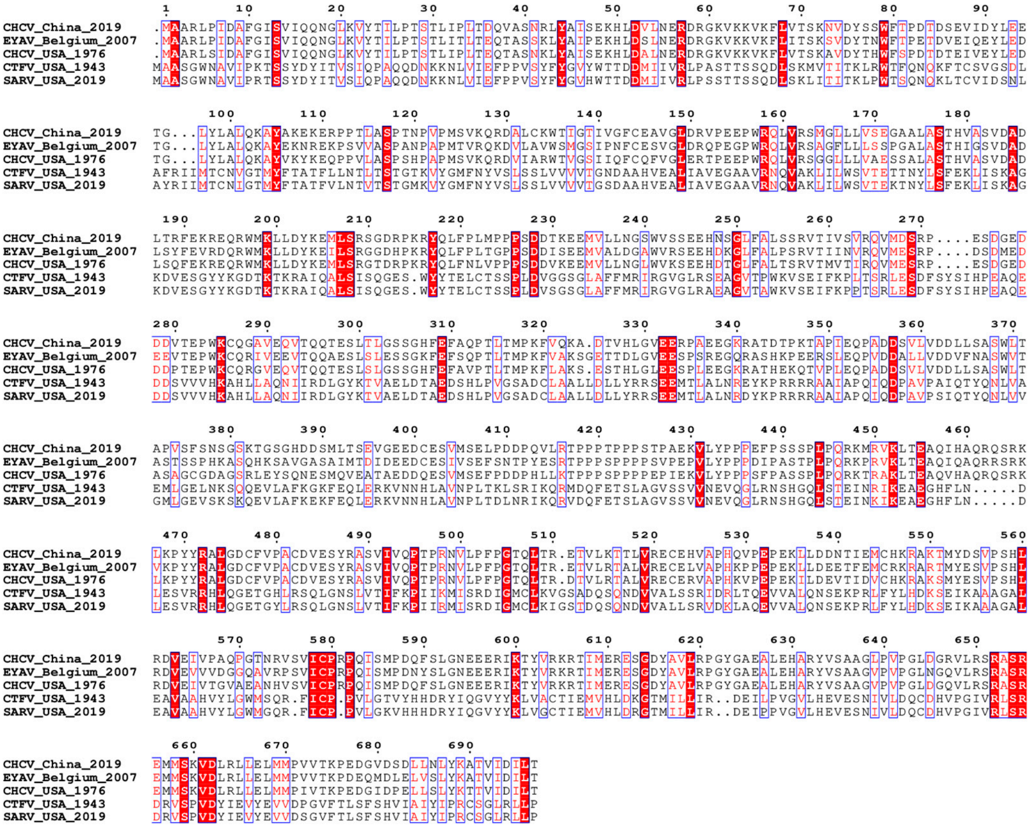

* CHCV, California hare coltivirus; CTFV, Colorado tick fever coltivirus; EYAV, Eyach virus; SARV, Salmon River virus.

Figure S8. Amino acid alignment of VP7 between CHCV_China and the representative *Coltivirus* in *Spinareoviridae*.

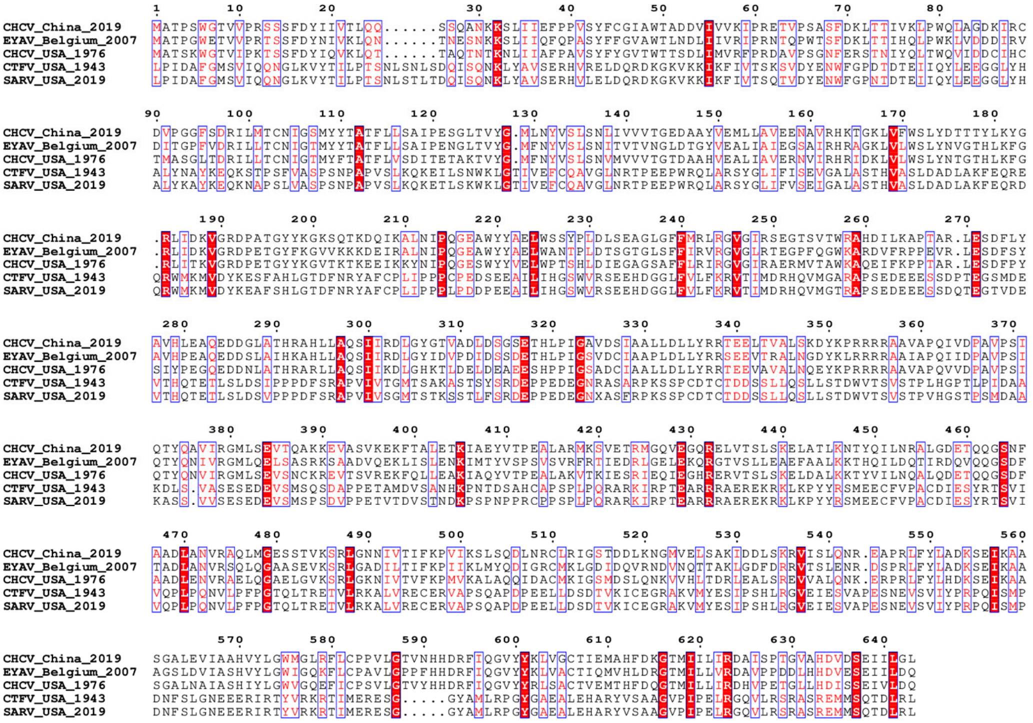

* CHCV, California hare coltivirus; CTFV, Colorado tick fever coltivirus; EYAV, Eyach virus; SARV, Salmon River virus.

Figure S9. Amino acid alignment of VP8 between CHCV_China and the representative *Coltivirus* in *Spinareoviridae*.

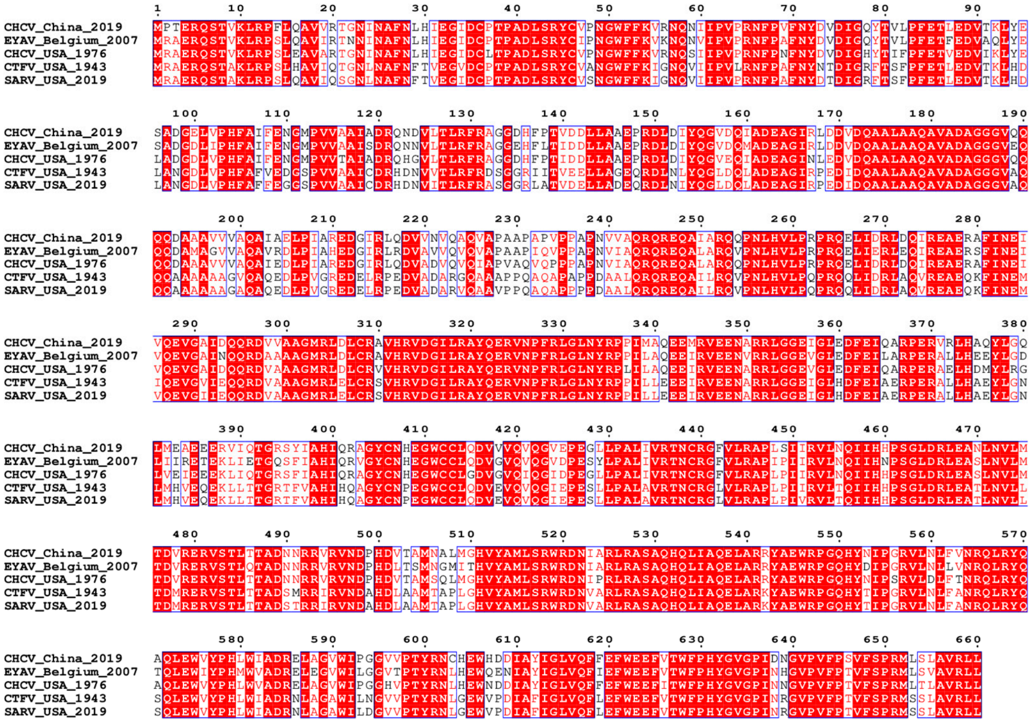

* CHCV, California hare coltivirus; CTFV, Colorado tick fever coltivirus; EYAV, Eyach virus; SARV, Salmon River virus.

Figure S10. Amino acid alignment of VP9 between CHCV_China and the representative *Coltivirus* in *Spinareoviridae*.

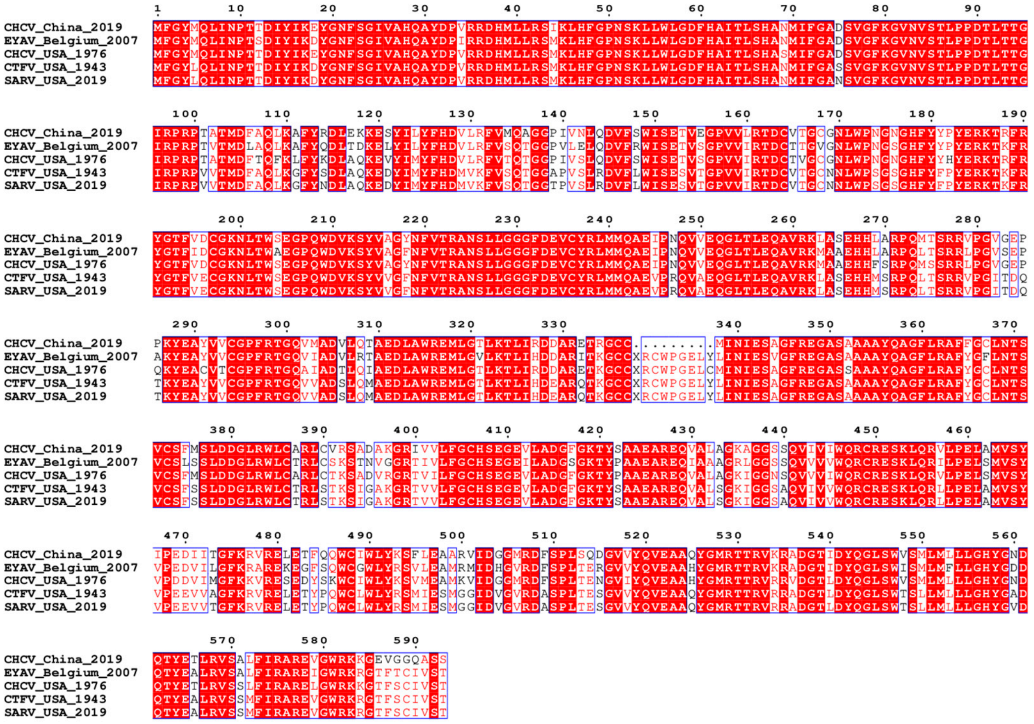

* CHCV, California hare coltivirus; CTFV, Colorado tick fever coltivirus; EYAV, Eyach virus; SARV, Salmon River virus.

Figure S11. Amino acid alignment of VP9' between CHCV_China and the representative *Coltivirus* in *Spinareoviridae*.

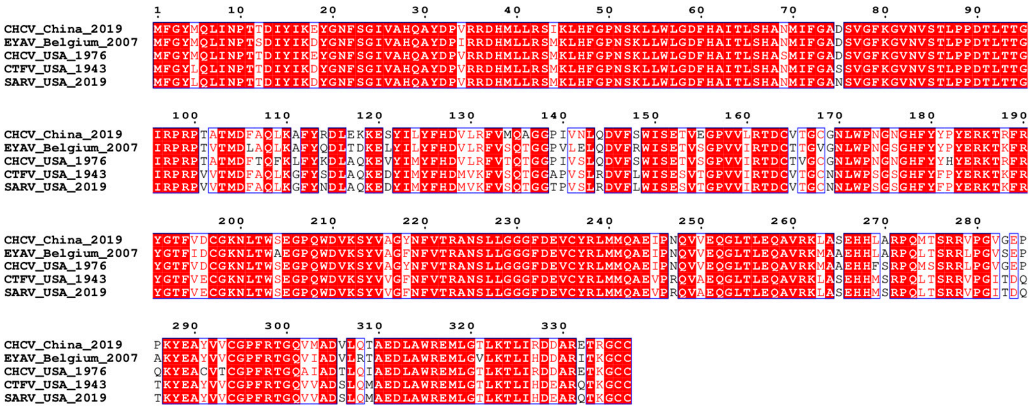

* CHCV, California hare coltivirus; CTFV, Colorado tick fever coltivirus; EYAV, Eyach virus; SARV, Salmon River virus.

Figure S12. Amino acid alignment of VP10 between CHCV_China and the representative *Coltivirus* in *Spinareoviridae*.

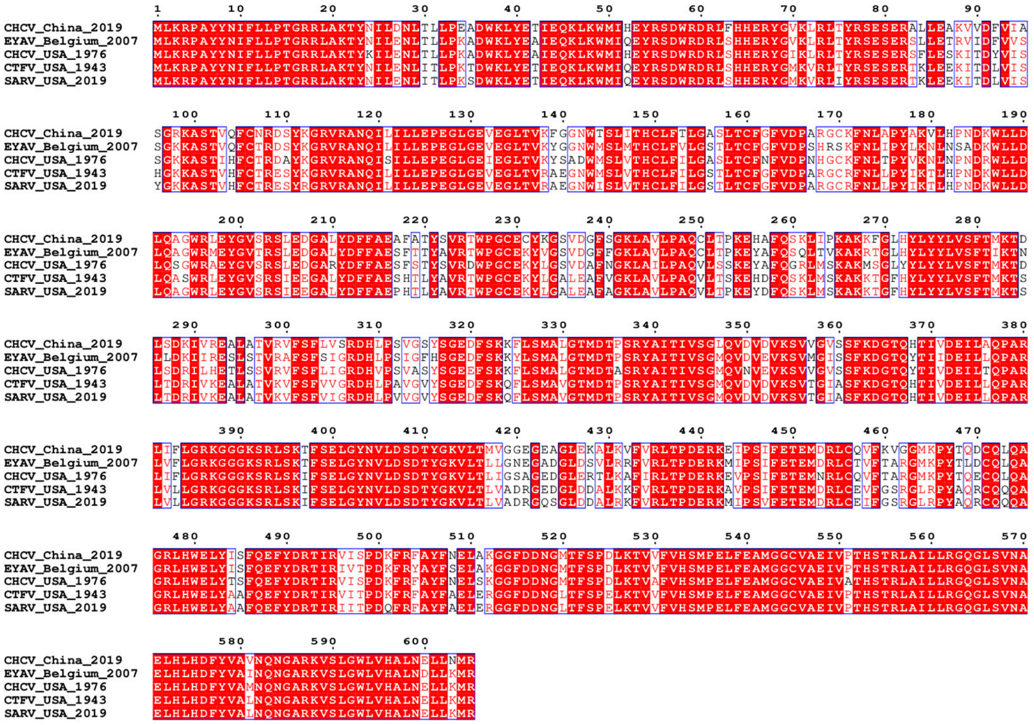

* CHCV, California hare coltivirus; CTFV, Colorado tick fever coltivirus; EYAV, Eyach virus; SARV, Salmon River virus.

Figure S13. Amino acid alignment of VP11 between CHCV_China and the representative *Coltivirus* in *Spinareoviridae*.

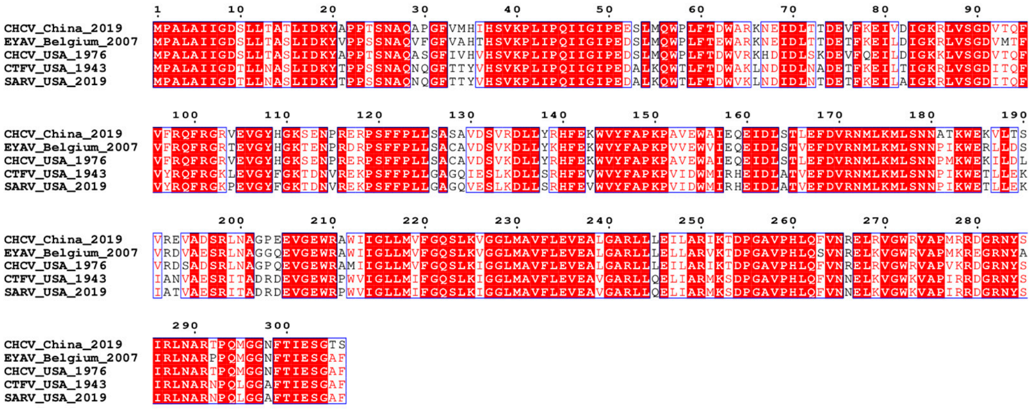

* CHCV, California hare coltivirus; CTFV, Colorado tick fever coltivirus; EYAV, Eyach virus; SARV, Salmon River virus.

Figure S14. Amino acid alignment of VP12 between CHCV_China and the representative *Coltivirus* in *Spinareoviridae*.

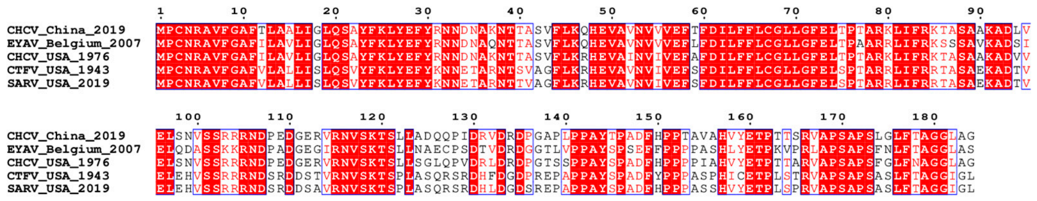

* CHCV, California hare coltivirus; CTFV, Colorado tick fever coltivirus; EYAV, Eyach virus; SARV, Salmon River virus.

Figure S15. Phylogenetic analysis of California hare Coltivirus_China for the deduced amino acid sequences (VP2-VP12).

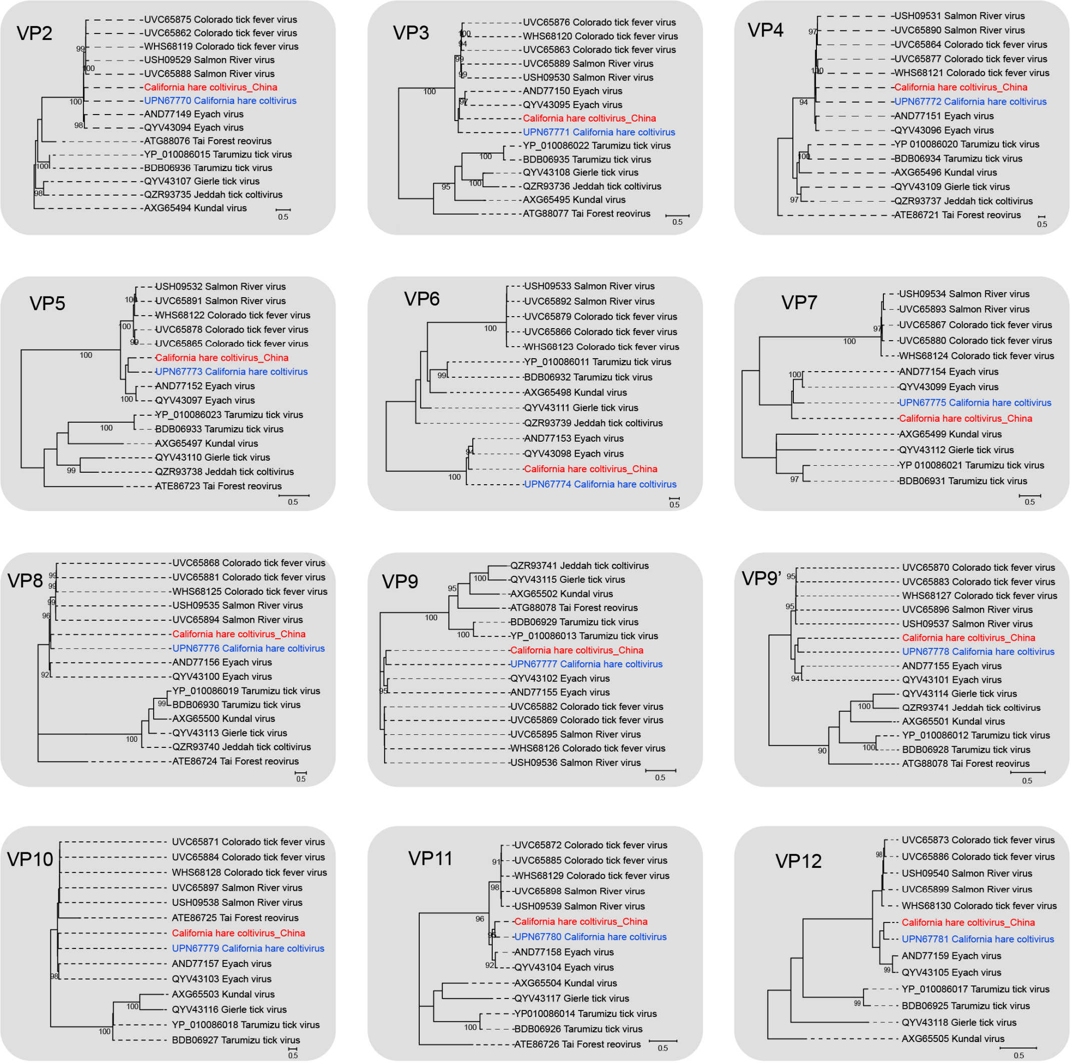

Phylogeny of deduced amino acid sequences (VP2-VP12) in the genus *Coltivirus*. Branch lengths are measured by a scale bar. California hare Coltivirus_China identified in this study is labeled by red front, and the blue represents the previously described virus.

Figure S16. Similarity plot of California hare Coltivirus_China and other representative viruses in Seg1-Seg12. Potential recombination events within viral genomes and possible recombination breakpoints were detected through Simplot (v3.5.1) and RDP (v4.97).

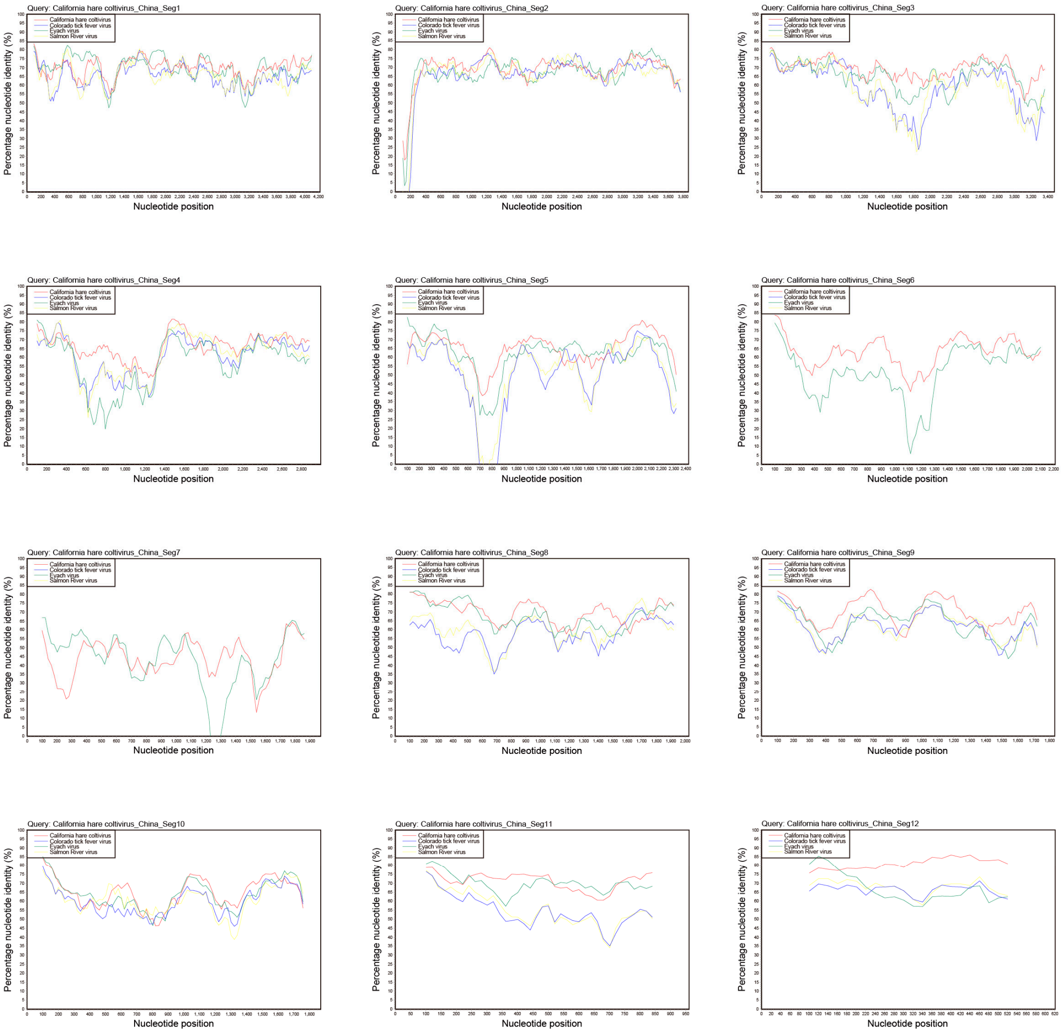

Table S1. **Primers for identifying species of ticks.**

Primer	Sequence (5'-3')	Size (bp)	Target Gene
ixodids-tick-F	CCGGTCTGAACTCAGATCAAGT	460	mitochondrial 16S rDNA
ixodids-tick-R	GCTCAATGATTTTTTAAATTGCTGT	460	mitochondrial 16S rDNA

Table S2. Viruses in *Spinareoviridae* used for phylogenetic analysis based on RNA-dependent RNA polymerase (RdRp) gene in the study.

Genera	Species	RdRp accession
Aquareovirus	chum salmon reovirus	AAL31497
	Green River chinook virus	AHJ14802
	golden shiner reovirus	AAM92745
	grass carp reovirus	AGG53846
	Scophthalmus maximus reovirus	ADZ31977
	American grass carp reovirus	ABV01040
Coltivirus	Colorado tick fever coltivirus_USA: California	WHS68118
	Colorado tick fever coltivirus_USA: Colorado	UVC65861
	Colorado tick fever coltivirus_USA: Wyoming	UVC65874
	Eyach virus_Belgium	QYV43093
	Eyach virus_France	AND77148
	Kundal virus_India	AXG65493
	Tai Forest reovirus_Cote d'Ivoire	ATG88075
	Tarumizu tick virus_Japan	YP_010086016
	Tarumizu tick virus_Japan	BDB06937
	California hare Coltivirus_USA: California	UPN67769
	Gierle tick virus_Belgium	QYV43106
	Jeddah tick coltivirus_Saudi Arabia	QZR93734
	Salmon River virus_USA: Idaho	USH09528
	Salmon River virus_USA: Oregon	UVC65887
Cypovirus	Lymantria dispar cypovirus 1	AAK73521
	Bombyx mori cypovirus 1	ALL27236
	Dendrolimus punctatus cypovirus 1	AAN46860
	Lymantria dispar cypovirus 14	AAK73088
	Choristoneura fumiferana cypovirus 16	ACA53380
	Inachis io cypovirus 2	AHJ14792
	Heliothis armigera cypovirus 5	ABV04400
	Orgyia pseudotsugata cypovirus 5	AHJ14782
Dinovernavirus	Aedes pseudoscutellaris reovirus	AAZ94069
Fijivirus	Fiji disease virus	AAK40249
	Maize rough dwarf virus	ANG56321
	Mal de Rio Cuarto virus	AAO73182
	Nilaparvata lugens reovirus	BAA08542
	Rice black streaked dwarf virus	CAC82519
	Southern rice black-streaked dwarf virus	CBH31251
Idnoreovirus	Operophtera brumata reovirus	ABB17205
Mycoreovirus	Cryphonectria parasitica mycoreovirus 1	AAP45577
	Rosellinia necatrix mycoreovirus 3	BAC98431
Orthoreovirus	avian orthoreovirus chicken isolate	CBX25023
	Muscovy duck reovirus goose isolate classical	AHL21592

	Muscovy duck reovirus novel	AFV52269
	Muscovy duck reovirus goose isolate novel	AFQ62078
	baboon orthoreovirus	AEK86189
	Broome reovirus	ACU68600
	Mahlapitsi virus	AMU04171
	mammalian orthoreovirus 3	ADJ00316
	mammalian orthoreovirus 1	ANJ21365
	mammalian orthoreovirus 2	AGG40205
	Nelson Bay orthoreovirus	AEQ49374
	Pycnonotidae orthoreovirus	BAQ19493
	tvarminne orthoreovirus	AHW40447
	piscine orthoreovirus	AGR44268
	reptilian orthoreovirus bush viper	AHL26961
	chelonian orthoreovirus	AOM63684
Oryzavirus	Rice ragged stunt virus	AAC36456
Outgroup	Banna virus	AAF77631

Table S3. Viruses in *Coltivirus* used for phylogenetic analysis based on VP2-VP12 gene in the study.

Species	VP2	VP3	VP4	VP5	VP6	VP7	VP8	VP9	VP9’	VP10	VP11	VP12
Colorado tick fever coltivirus	WHS68119	WHS68120	WHS68121	WHS68122	WHS68123	WHS68124	WHS68125	WHS68126	WHS68127	WHS68128	WHS68129	WHS68130
Colorado tick fever coltivirus	UVC65862	UVC65863	UVC65864	UVC65865	UVC65866	UVC65867	UVC65868	UVC65869	UVC65870	UVC65871	UVC65872	UVC65873
Colorado tick fever coltivirus	UVC65875	UVC65876	UVC65877	UVC65878	UVC65879	UVC65880	UVC65881	UVC65882	UVC65883	UVC65884	UVC65885	UVC65886
Eyach virus	QYV43094	QYV43095	QYV43096	QYV43097	QYV43098	QYV43099	QYV43100	QYV43102	QYV43101	QYV43103	QYV43104	QYV43105
Eyach virus	AND77149	AND77150	AND77151	AND77152	AND77153	AND77154	AND77156	AND77155	-	AND77157	AND77158	AND77159
Kundal virus	AXG65494	AXG65495	AXG65496	AXG65497	AXG65498	AXG65499	AXG65500	AXG65502	AXG65501	AXG65503	AXG65504	AXG65505
Tai Forest reovirus	ATG88076	ATG88077	ATE86721	ATE86723	-	-	ATE86724	ATG88078	-	ATE86725	ATE86726	-
Tarumizu tick virus	YP_010086015	YP_010086022	YP_010086020	YP_010086023	YP_010086011	YP_010086021	YP_010086019	YP_010086013	YP_010086012	YP_010086018	YP_010086014	YP_010086017
Tarumizu tick virus	BDB06936	BDB06935	BDB06934	BDB06933	BDB06932	BDB06931	BDB06930	BDB06929	BDB06928	BDB06927	BDB06926	BDB06925
California hare Coltivirus	UPN67770	UPN67771	UPN67772	UPN67773	UPN67774	UPN67775	UPN67776	UPN67777	UPN67778	UPN67779	UPN67780	UPN67781
Gierle tick virus	QYV43107	QYV43108	QYV43109	QYV43110	QYV43111	QYV43112	QYV43113	QYV43115	QYV43114	QYV43116	QYV43117	QYV43118
Jeddah tick coltivirus	QZR93735	QZR93736	QZR93737	QZR93738	QZR93739	-	QZR93740	QZR93741	QZR93741	-	-	-
Salmon River virus	USH09529	USH09530	USH09531	USH09532	USH09533	USH09534	USH09535	USH09536	USH09537	USH09538	USH09539	USH09540
Salmon River virus	UVC65888	UVC65889	UVC65890	UVC65891	UVC65892	UVC65893	UVC65894	UVC65895	UVC65896	UVC65897	UVC65898	UVC65899

Table S4. Nucleotide similarity (%) between California hare Coltivirus_China and the representative *Coltivirus* in *Spinareoviridae*.

Segments	Virus species	CHCV_China	CHCV_USA	CTFV	EYAV
Seg1					
	CHCV_USA	76.68	***	***	***
	CTFV	73.89	76.00	***	***
	EYAV	76.67	76.27	75.57	***
	SARV	73.94	75.51	88.66	74.27
Seg2					
	CHCV_USA	75.63	***	***	***
	CTFV	75.54	76.77	***	***
	EYAV	75.58	75.28	75.64	***
	SARV	75.57	75.70	88.03	75.93
Seg3					
	CHCV_USA	76.33	***	***	***
	CTFV	71.43	71.84	***	***
	EYAV	74.01	74.24	71.12	***
	SARV	71.28	72.08	85.86	71.07
Seg4					
	CHCV_USA	75.17	***	***	***
	CTFV	72.92	71.87	***	***
	EYAV	71.64	73.11	72.10	***
	SARV	73.25	72.46	87.80	72.58
Seg5					
	CHCV_USA	74.29	***	***	***
	CTFV	68.86	69.54	***	***
	EYAV	73.56	71.18	69.22	***
	SARV	70.03	69.63	88.41	69.86
Seg6					
	CHCV_USA	73.48	***	***	***
	CTFV	-	-	***	***
	EYAV	69.15	71.40	-	***
	SARV	-	-	86.92	-
Seg7					
	CHCV_USA	65.48	***	***	***
	CTFV	-	-	***	***
	EYAV	68.11	73.37	-	***
	SARV	-	-	86.93	-
Seg8					
	CHCV_USA	77.00	***	***	***
	CTFV	70.44	70.96	***	***
	EYAV	76.04	74.88	69.04	***
	SARV	71.73	70.34	87.13	68.85
Seg9					
	CHCV_USA	77.34	***	***	***
	CTFV	72.55	74.10	***	***
	EYAV	73.85	76.14	72.77	***
	SARV	72.37	74.89	94.06	73.78
Seg10					
	CHCV_USA	74.63	***	***	***
	CTFV	72.02	73.97	***	***
	EYAV	74.22	75.81	73.51	***
	SARV	72.86	73.82	89.10	74.14
Seg11					

	CHCV_USA	79.24	***	***	***
	CTFV	71.16	71.90	***	***
	EYAV	78.11	78.74	71.96	***
	SARV	71.68	71.95	91.72	72.48
Seg12					
	CHCV_USA	83.79	***	***	***
	CTFV	75.13	76.05	***	***
	EYAV	77.10	77.83	72.13	***
	SARV	76.87	76.15	96.99	71.85

* CHCV, California hare coltivirus; CTFV, Colorado tick fever coltivirus; EYAV, Eyach virus; SARV, Salmon River virus.

Table S5. Amino acid similarity (%) between California hare Coltivirus_China and the representative *Coltivirus* in *Spinareoviridae*.

	Virus species	CHCV_China	CHCV_USA	CTFV	EYAV
VP1					
	CHCV_USA	90.46	***	***	***
	CTFV	87.69	87.81	***	***
	EYAV	89.32	89.55	86.41	***
	SARV	87.62	87.39	97.91	86.55
VP2					
	CHCV_USA	91.36	***	***	***
	CTFV	89.04	89.29	***	***
	EYAV	90.63	90.26	89.93	***
	SARV	89.23	89.05	98.90	89.60
VP3					
	CHCV_USA	88.38	***	***	***
	CTFV	78.75	79.78	***	***
	EYAV	84.91	84.35	77.33	***
	SARV	78.75	80.12	95.60	77.24
VP4					
	CHCV_USA	85.66	***	***	***
	CTFV	81.24	80.43	***	***
	EYAV	79.94	80.53	78.87	***
	SARV	81.85	80.82	97.86	79.75
VP5					
	CHCV_USA	84.02	***	***	***
	CTFV	75.77	72.80	***	***
	EYAV	81.47	80.13	73.84	***
	SARV	75.20	72.67	95.47	73.71

VP6					
	CHCV_USA	80.52			
	CTFV	-	-		
	EYAV	73.82	74.96	-	
	SARV	-	-	92.83	-
VP7					
	CHCV_USA	64.59	***	***	***
	CTFV	-	-	***	***
	EYAV	64.59	61.98	-	***
	SARV	-	-	91.96	-
VP8					
	CHCV_USA	88.94	***	***	***
	CTFV	75.00	75.00	***	***
	EYAV	85.61	85.15	73.64	***
	SARV	76.36	76.36	96.36	75.15
VP9					
	CHCV_USA	90.50	***	***	***
	CTFV	84.87	83.68	***	***
	EYAV	89.02	87.54	84.57	***
	SARV	84.87	83.68	99.11	84.57
VP9’					
	CHCV_USA	86.87	***	***	***
	CTFV	82.32	82.72	***	***
	EYAV	83.84	84.55	83.06	***
	SARV	82.49	82.72	99.17	83.06
VP10					
	CHCV_USA	84.13	***	***	***
	CTFV	82.98	80.83	***	***
	EYAV	85.29	82.81	81.32	***

	SARV	81.82	79.17	96.53	81.32
VP11					
	CHCV_USA	90.49	***	***	***
	CTFV	73.11	73.94	***	***
	EYAV	86.89	86.36	75.57	***
	SARV	73.68	74.59	98.05	75.90
VP12					
	CHCV_USA	88.11	***	***	***
	CTFV	71.58	70.49	***	***
	EYAV	75.96	77.05	64.48	***
	SARV	73.22	73.08	95.14	67.03

* CHCV, California hare coltivirus; CTFV, Colorado tick fever coltivirus; EYAV, Eyach virus; SARV, Salmon River viru
